# Antibiotic Resistance, Core-Genome and Protein Expression in IncHI1 Plasmids in *Salmonella* Typhimurium

**DOI:** 10.1093/gbe/evw105

**Published:** 2016-05-05

**Authors:** Tereza Kubasova, Darina Cejkova, Jitka Matiasovicova, Zuzana Sekelova, Ondrej Polansky, Matej Medvecky, Ivan Rychlik, Helena Juricova

**Affiliations:** ^1^Veterinary Research Institute, Hudcova 70, Brno 621 00, Czech Republic

**Keywords:** IncHI1 plasmids, comparative genomic, proteomics

## Abstract

Conjugative plasmids from the IncHI1 incompatibility group play an important role in transferring antibiotic resistance in *Salmonella* Typhimurium. However, knowledge of their genome structure or gene expression is limited. In this study, we determined the complete nucleotide sequences of four IncHI1 plasmids transferring resistance to antibiotics by two different next generation sequencing protocols and protein expression by mass spectrometry. Sequence data including additional 11 IncHI1 plasmids from GenBank were used for the definition of the IncHI1 plasmid core-genome and pan-genome. The core-genome consisted of approximately 123 kbp and 122 genes while the total pan-genome represented approximately 600 kbp. When the core-genome sequences were used for multiple alignments, the 15 tested IncHI1 plasmids were separated into two main lineages. GC content in core-genome genes was around 46% and 50% in accessory genome genes. A multidrug resistance region present in all 4 sequenced plasmids extended over 20 kbp and, except for *tet(B)*, the genes responsible for antibiotic resistance were those with the highest GC content. IncHI1 plasmids therefore represent replicons that evolved in low GC content bacteria. From their original host, they spread to *Salmonella* and during this spread these plasmids acquired multiple accessory genes including those coding for antibiotic resistance. Antibiotic-resistance genes belonged to genes with the highest level of expression and were constitutively expressed even in the absence of antibiotics. This is the likely mechanism that facilitates host cell survival when antibiotics suddenly emerge in the environment.

## Introduction

Acquired antibiotic resistance is continuously increasing among bacterial pathogens including *Salmonella enterica* (Glenn et al. 2011). Although multiple genetic elements are involved in the spread of antibiotic resistance in bacterial populations, conjugative plasmids belong among the most common vehicles.

Conjugative plasmids are commonly classified into incompatibility groups based on their origin of replication (Carattoli 2009). The most frequent conjugative plasmids in *Enterobacteriaceae* can be classified into IncI, IncFI, IncN and IncH incompatibility groups and all of these plasmids play important roles in transferring antibiotic resistance in different serovars of *S. enterica* (Rychlik et al. 2006; Hradecka et al.2008; Suzuki et al. 2010). IncHI1 plasmids have been intensively studied due to their high-molecular weight and temperature-dependent conjugation. Their temperature-dependent conjugation (optimum at 22–30 °C) indicates that the origin of IncHI1 plasmids can be traced back to microbial populations in water and soil environments (Maher and Taylor 1993). IncHI1 plasmids were also found in human adapted bacterial pathogens, for example, in *S. enterica* serovar Typhi (Phan et al. 2009, Holt et al. 2011). Another characteristic of these plasmids is their high-molecular weight. IncHI1 plasmids are commonly larger than 180 kbp and the size is due to the incorporation of genes responsible for resistance to antibiotics or heavy metals, utilization of alternative carbon sources or production of antimicrobial toxins (Dolejska et al. 2013). Resistance genes are often parts of multiple mobile elements inserted in IncHI1 plasmids, for example, Tn10 with tetracycline resistance or Tn9/Tn21 composite transposon encoding resistance to chloramphenicol and mercury (Holt et al. 2007).

Although more than 10 different IncHI1 plasmids have already been sequenced (Sherburne et al. 2000; Parkhill et al. 2001; Holt et al. 2007; Ogura et al. 2009; Ong et al. 2012; Dolejska et al. 2013, 2014; Conlan et al. 2014), knowledge about their genetic structure is much less known in comparison with plasmids from other incompatibility groups such as IncI plasmids (Takahashi et al. 2011; Kubasova et al. 2014). In our previous study, we characterized four IncHI1 plasmids of *S. enterica* serovar Typhimurium (*S.* Typhimurium) in respect to conjugation and antibiotic resistance (Hradecka et al. 2008). In the present study, we determined their complete nucleotide sequences and compared them among themselves and also with other IncHI1 plasmids deposited in GenBank. This analysis enabled the definition of the core-genome of the ancestral IncHI1 plasmid backbone likely circulating in bacterial populations in the era before the widespread clinical use of antibiotics, as well as the pan-genome, that is, all different sequences which have ever inserted into the IncHI1 backbone sequence. Finally, we determined the protein expression profile from all four newly sequenced plasmids. We concluded that the core-genome of IncHI1 plasmids is defined by 122 genes and that 2 different IncH1 plasmid lineages circulate in bacterial populations.

## Materials and Methods

### Bacterial Strains

*Salmonella enterica* serovar Typhimurium strain 21G7 harboring plasmid pB71, strain 21G5 harboring plasmid p109-9, strain 21G6 harboring pF8475 and strain 21I5 harboring plasmid p8025 were used in this study (Hradecka et al. 2008). Before sequencing, all plasmids were transferred by conjugation to plasmid free *S.* Typhimurium F98. Bacterial strains were routinely grown at 24 °C in Luria-Bertani (LB) broth. Because all four plasmids coded for tetracycline resistance, LB broth was supplemented with tetracycline (12 μg/ml) to maintain positive selection of the plasmid positive cells unless otherwise stated.

### DNA Purification and Sequencing

One milliliter of overnight culture in LB was pelleted by centrifugation at 14,000 × g for 1 min and total DNA was purified using DNeasy Tissue Kit following the manufacturer’s instructions (Qiagen, Germany). Sequencing libraries were prepared from 500 and 1ng of total bacterial DNA using the 454 Rapid Library kit (Roche, Switzerland) and Nextera XT DNA Sample Preparation kit (Illumina, USA), respectively. Sequencing reactions were performed using GS Junior Titanium sequencing chemistry and a GS Junior 454 sequencer (Roche) or MiSeq Reagent Kit v3 and MiSEQ 2000 apparatus (Illumina), always strictly following the manufacturer’s instructions.

### De novo Assembly and Gap Closure

Individual reads obtained from 454 pyrosequencing were assembled by Newbler (Roche). To estimate the order of assembled plasmid contigs, the contigs were aligned against plasmids R27 [GenBank: AF250878], pMAK1 [GenBank: AB366440], pHCM1 [GenBank: AL513383] and pAKU_1 [GenBank: AM412236] using commercial proprietary assembler Seqman from the DNASTAR package (Lasergene, USA). In the next step, primers flanking the predicted gaps between ordered contigs were designed and used in PCR. PCR was carried out using PCR Master Mix (Qiagen) for gaps predicted to be <2 kbp or LA DNA Polymerase Mix (Top-Bio, Czech Republic) for gaps predicted to be >2 kbp. PCR cycling conditions consisted of initial denaturation at 95 °C for 5 min, followed by 30 cycles at 94 °C for 40 s, 55 °C for 40 s and primer extension at 72 °C. Primer extension in standard PCR lasted for 60 s while this time was extended to 5 min in the case of LA DNA polymerase. The final extension step was performed at 72 °C for 10 min. Amplified PCR products were separated by electrophoresis on a 1.0% agarose gel, purified using QIAquick PCR Purification kit (Qiagen) and sequenced using a commercial service with dideoxy terminator sequencing chemistry (https://www.gatc-biotech.com).

Illumina sequencing was used to improve plasmid sequences, especially in homopolymeric regions. The quality trimming of the raw reads was performed using TrimmomaticPE (Version 0.32; Bolger et al. 2014). The chromosomal reads were filtered out against the reference *S.* Typhimurium F98 sequence using the BWA-MEM algorithm (Li 2013) and the plasmid reads were aligned by *de novo* assembler IDBA_UD (Version 1.1.1; Peng et al. 2012). Discrepancies between 454 and Illumina were found only in homopolymeric regions that were automatically corrected according to the Illumina reads. Detailed information on sequencing and assembly statistics is shown in supplementary table S1, Supplementary Material online.

### Annotation

All plasmid sequences were first automatically annotated using BioNumerics software version 7.1. (Applied Maths, Belgium). GenBank entries of R27 [GenBank: AF250878], pMAK1 [GenBank: AB366440], pHCM1 [GenBank: AL513383], pAKU_1 [GenBank: AM412236] and pNDM-CIT [GenBank: JX182975] were used as sources for annotation. The automatic annotation was then manually verified. Maps of multidrug resistance (MDR) regions were generated using SnapGene^®^ 2.5 (GSL Biotech LLC, USA). The complete nucleotide sequence of plasmids pB71, p109-9, pF8475 and p8025 have been deposited in the GenBank under accession numbers KP899806, KP899805, KP899804 and KP899803, respectively.

### Definition of Core-Genome and Pan-Genome Sequence

All 4 examined plasmids from this study and 11 additional IncHI1 plasmids (pHCM1 [AL513383], pAKU_1 [AM412236], pEQ1 [KF362121], pEQ2 [KF362122], pO111 [AP010961], pPstx_12_1 [CP003279], pPsP-75c [CP009869], p34983 [CP010380], pR27 [AF250878], pNDM-CIT [JX182975], pMAK1 [AB366440]) were used for the definition of the core-genome and pan-genome. For the construction of the pan-genome, the strategy based on iterative pan-genome construction has been used (Laing et al. 2010; Tomida et al. 2013). Briefly, p109-9 plasmid sequence was used as the first reference sequence and compared with pB71 DNA using cross_match software from the Phrap package (http://www.phrap.org/phredphrapconsed.html). Unique regions from pB71 were added to the p109-9 plasmid sequence thus creating the second reference sequence. In the next step, the second reference sequence was used for comparison with the pF8475 sequence. These steps were iterated until all 15 genomes were compared and the last (15th) reference genome was considered as the pan-genome sequence. The core-genome sequence was determined in a similar way, however, instead of adding unique regions of individual genomes, only the shared core between the compared genomes was retained at each iterative step.

### Comparative Analysis of Plasmids

The complete sequences of 15 different plasmids and core-genome sequences were aligned to the pan-genome sequences using the BLASTn algorithm implemented in the BRIG (BLAST ring image generator) program (Version 0.95; Alikhan et al. 2011). The same program was also employed for visualizing the comparison. The collinearity and rearrangement of plasmid sequences were analyzed by WebACT software (Abbott et al. 2007).

To compare the plasmids based on the on the presence/the absence of proteins, Prokka annotation (Seemann 2014) was used for the protein prediction in all 15 examined plasmids. The predicted proteins were aligned and clustered using CD-HIT (Fu et al. 2012) and homologous proteins were defined as those that shared over 40% sequence identity over 80% of their length (Pearson 2013). Binary matrix showing the presence or the absence of particular protein cluster in examined plasmids was generated and complete linkage cluster analysis based on the matrix was generated in R Studio.

To estimate the phylogenetic relatedness among plasmids, two different strategies were applied. The first phylogenetic analysis included 15 plasmid core-genome backbones. The core backbone regions were extracted from individual plasmid sequences, ordered, inverted and complemented if appropriate, and concatenated. The alternative comparison was performed using only the sequences of genes proposed for IncHI1 plasmid multilocus sequence typing (pMLST), that is, orthologs of the genes HCM1.043, 064, 099, 116, 178ac and 259 (Phan et al. 2009). Complete coding sequences (CDS), sequences generated from the first and second codon positions, and sequences generated only from the third codon positions were used and concatenated. Concatenated sequences were then aligned by the CLUSTALW program (Version 1.4) followed by visual inspection of the aligned sequences (Thompson et al. 1994). The phylogenetic trees were produced with a maximum likelihood (ML) algorithm incorporated in PhyML 3.0 software based on the best fitting nucleotide substitution models (Guindon 2010). The substitution models were estimated by jModelTest (supplementary table S2, Supplementary Material online; Darriba et al. 2012). Trees were constructed using optimized topology, the branch lengths and rate parameters option with BIONJ tree used as a starting tree. Different substitution rate categories were applied as predicted by jModelTest (supplementary table S2, Supplementary Material online). The reliability of the tree branches was computed by an approximate-likelihood ratio test (aLRT), a SH-like statistical support (Anisimova and Gascuel 2006). Predicted trees were visualized by FigTree viewer (http://tree.bio.ed.ac.uk/software/figtree/).

### The Origin of Plasmid Sequences

KmerFinder 2.0 was used to estimate the original host organism from which the plasmid sequences were derived (Hasman et al. 2014; Larsen et al. 2014). Because the core and the accessory genome could have been of different origin accessory and core-genome regions of pB71, p109-9, pF8475 and p8025 plasmids were concatenated and analyzed separately.

### Protein Mass Spectrometry (MS)

In total, three independent experiments were performed. In the first experiment, bacterial cells were grown at 24 °C for 20 h in LB broth supplemented with tetracycline (12 μg/ml), the conditions at which we expected maximal plasmid gene expression due to the optimal plasmid conjugation at this temperature. In the second experiment, bacterial cells were grown in antibiotic-free LB broth at 24 °C for 7 h and in the last experiment, bacterial cells were grown in antibiotic-free LB broth at 24 °C for 20 h followed by a 6-day incubation at 4 °C, to test whether some of the plasmid encoded genes might be specifically expressed at stressed condition in stationary phase of growth and decreased temperature. In all experiments, cells from 1 ml of bacterial culture were pelleted by centrifugation for 1 min at 14,000 × g, lysed with TRI reagent and purified according to the manufacturer’s recommendations (MRC). Dried protein pellets were dissolved in 300 µl of 8 M urea and processed by the modified Filter Aided Sample Preparation method (Wisniewski et al. 2009) using Vivacon 500 with a 10-kDa molecular weight cut off (Sartorius Stedim Biotech). Dissolved proteins were washed twice with 100 µl of 8 M urea and reduced by adding 100 µl of 10 mM dithiothreitol. After reduction, the proteins were incubated in 100 µl of 50 mM iodoacetamide and washed twice with 100 µl of 25 mM triethylammonium bicarbonate. Trypsin was used at 1:50 ratio as a digestive enzyme.

Liquid chromatography–tandem mass spectrometry (LC–MS/MS) analysis was performed by using a Dionex UltiMate 3000 RSLCnano system connected to Obitrap VelosPro mass spectrometer. Injected samples were desalted and preconcentrated during the first 4 min of LC–MS/MS run on the Acclaim PepMap C18 trapping column (2 cm × 75 µm, 3 µm particles) at a flow rate 5 µl/min using loading mobile phase consisting of 0.1% formic acid in 98/2H_2_O/acetonitrile (vol/vol). Chromatographic separation was performed on EASY-Spray C18 separation column (50 cm × 75 µm, 3 µm particles) using 200 nl/min flow rate of Solvent A (0.1% formic acid) and Solvent B (0.1% formic acid in 20/80H_2_O/ACN [vol/vol]) and 430 min long reverse-phase gradient with concentration of Solvent B increasing from 6% to 45%.

High resolution (30,000 full width at half maximum at 400 *m*/*z*) MS spectra were acquired for the 390–1700 *m*/*z* interval in the Orbitrap VelosPro analyzer. Low resolution MS/MS spectra were acquired in the Linear Ion Trap in a data-dependent manner. The top 10 precursors (in terms of abundance) were fragmented using collision-induced dissociation fragmentation at a normalized collision energy of 35. Data were analyzed using the Proteome Discoverer (v.1.4). MS/MS spectra identification was performed by SEQUEST search against annotated plasmid sequences. Precursor and fragment mass tolerance for searches were 10 ppm and 0.6 Da, respectively. Only peptides passing the false discovery rate ≤ 1% (Percolator) were considered.

## Results

### Complete Nucleotide Sequences

The sizes of the sequenced plasmids ranged from 190,730 bp (pB71; 46.24% GC) to 311,280 bp (p8025; 50.74% GC). The sizes of other two plasmids were 207,203 bp (p109-9; 46.76% GC), and 210,266 bp (pF8475; 47.51% GC). Totally, 175, 200, 208, and 291 genes were predicted for the sequenced plasmids pB71, p109-9, pF8475, and p8025, respectively. IncHI1 plasmids ranging from 180 to 290 kbp in size showed lower GC content (45.75–47.73% GC) whereas larger plasmids (>300 kbp) exhibited slightly higher GC content (50.74–51.52% GC).

### Regions Coding for Antibiotic and Heavy Metal Resistances

**Fig. 1.—:**
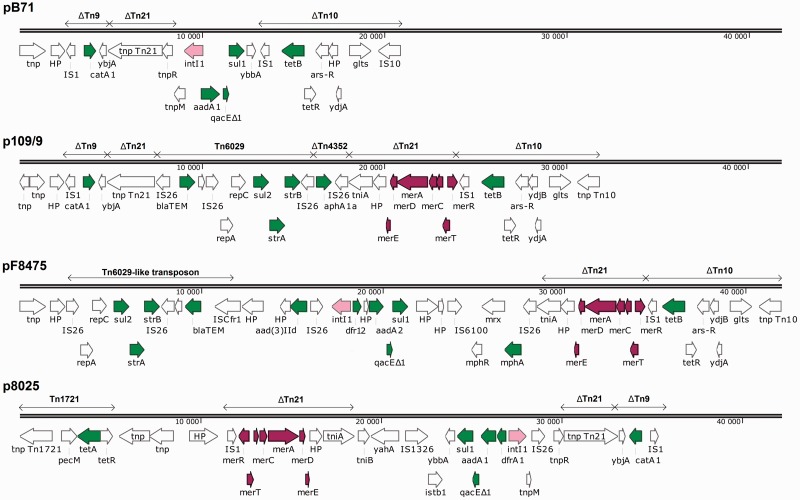
Genetic organization of the MDR regions in sequenced plasmids. Green, resistance genes; pink, integrase genes; violet, mercury-resistance genes. Numbers on the lines show the total length of MDR but do not correspond to individual plasmid coordinates.

The MDR region represented over 20 kbp of genetic information in each plasmid and encoded 5–11 resistance genes (fig. 1). All plasmids coded for tetracycline resistance. Plasmids pB71, p109-9, and pF8475 carried the *tet(B)* gene located in transposon Tn10 whereas plasmid p8025 harbored *tet(A)* as a part of Tn1721. *bla*_TEM_, *strA*, *strB*, and *sul2* genes comprised a single genetic element in plasmid pF8475 flanked by insertion of sequences IS26 and ISCfr1. A similar gene arrangement was found in p109-9 as a part of Tn6029 which was flanked by IS26 at both ends. Plasmids pB71, pF8475, and p8025 possessed Class 1 integrons that contained *aadA* and *dfrA* gene cassettes coding for resistance to streptomycin and trimethoprim, respectively (fig. 1).

The sequenced plasmids also encoded resistance to heavy metals. Mercury-resistance genes carried by Tn21 transposon were found as a part of MDR region in plasmids p109-9, pF8475, and p8025, always adjacent to an IS1 element. Plasmid p8025 coded also for arsenic and tellurium resistance and these genes were located outside the MDR.

### Determination of IncHI1 Plasmids Core-Genome and Pan-Genome

**Fig. 2.—:**
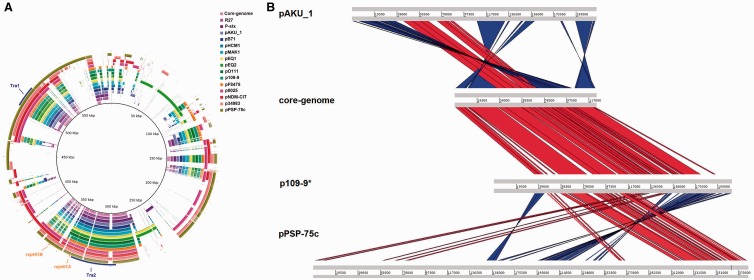
Comparison of IncHI1 plasmids. (*A*) BLASTn-based comparison of 15 IncHI1 plasmids resulted in the definition of IncHI1 core-genome and pan-genome sequences. The innermost ring represents pan-genome sequence whereas the remaining rings represent genomes of 15 plasmid sequences. Matches with an identity between 90% and 100% are colored. The outermost ring shows annotations in the most conserved regions: transfer regions (blue) and replication regions (orange). The image was generated by the BRIG software. (*B*) The collinearity and rearrangements of the core-genome regions. Red lines indicate regions of similarity in the same direction while blue lines indicate similar regions inverted from each other. The image was generated by the WebACT tool.

Next we compared the nucleotide sequences of four IncHI1 plasmids characterized in this study and additional 11 IncHI1 plasmid sequences available in GenBank and determined the core-genome and pan-genome of investigated IncHI1 plasmids (fig. 2*A*).

The plasmid pan-genome comprised of 597,956 bp while the core-genome was 123,370 bp. The core-genome comprised of 122 genes including genes for replication proteins (*repHIA*, *repHIB*) and 20 plasmid maintenance genes, 32 *tra* genes located in 2 separate clusters (Tra1 and Tra2), an IS1 element, and 67 hypothetical genes (fig. 2*A*). The pan-genome sequences comprised, in addition to the core-genome, genes carrying resistance to antibiotics and heavy metals, genes extending the metabolic capacity of plasmid host cells, for example, genes for citrate or fructooligosaccharide utilization, and hypothetical genes of currently unknown functions. Except for two plasmids, the remaining plasmids were collinear, that is, these followed exactly the same order of core-genome genes. The two exceptions included plasmids pAKU_1 and pPSP-75c. In plasmid pAKU_1, an inversion included *repHI1B* and Tra1 region while in pPSP-75c, an inversion included Tra1 region (fig. 2*B*). In all cases, IS1 transposase was adjacent to the inverted regions.

**Fig. 3.—:**
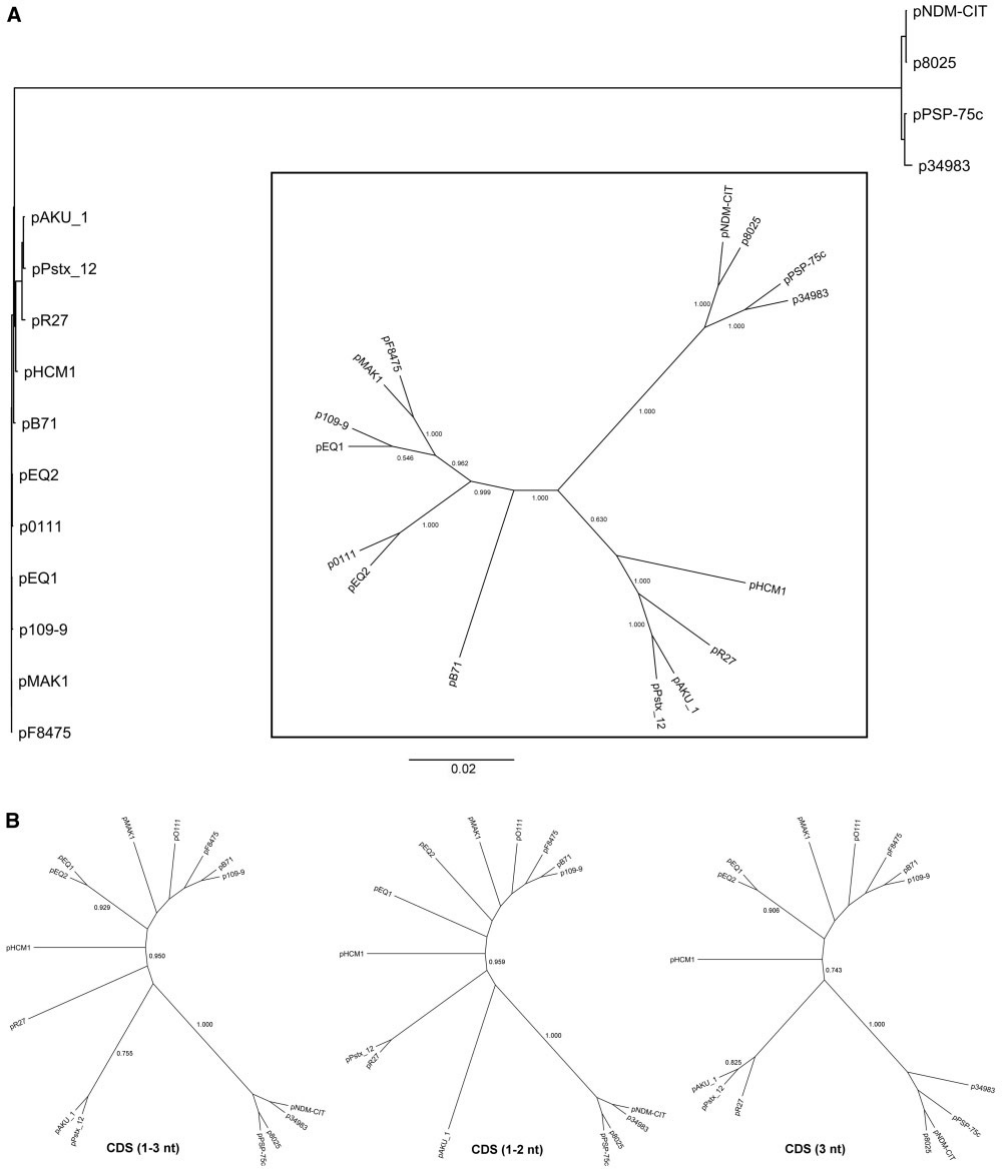
Phylogenetic relationship among 15 IncHI1 plasmids. (A) Phylogenetic phylogram constructed from core-genome sequences. Branch lengths are proportional to the number of nucleotide substitutions per position as indicated by the scale bar. An *inlet*, the same tree shown as an unrooted radial cladogram. (*B*) Unrooted radial cladograms generated from concatenated sequences of pMLST genes: CDS (1–3 nt), whole coding sequences; CDS (1–2 nt), sequences from the first and second codon positions; CDS (3 nt), sequences from the third codon positions. Numbers above the branches represent support values as determined by aLRT. For image clarity, support values are shown only in cladograms but not in phylogram.

The definition of the core-genome also enabled to characterize the evolutionary relationship of IncHI1 plasmids (fig. 3*A*). A constructed phylogenetic tree based on ML method resulted in two major clusters. The first cluster comprised p8025, pNDM-CIT, pPSP-75c, and p34983 plasmids and the second cluster was formed by all remaining IncHI1 plasmids. A closer look at the phylogenetic relationship of the plasmids forming the majority cluster indicated that these could be further subdivided into two subgroups. Because the phylogenetic relationship based on core-genome sequences can be affected by different mutation rates and different codon positions; we subsequently analyzed the plasmid relatedness using either concatenated sequences of pMLST typing genes (Phan et al. 2009), or separately for the whole CDS (1–3 nt), or the sequences generated from the first and second codon positions (CDS 1–2 nt), or sequences at the third codon positions (CDS 3 nt). Because HCM1.178ac and HCM1.259 genes were absent in p8025, pPSP-75c, pNDM-CIT, and P_stx_12_1 plasmids, sequences from only four concatenated genes (HCM1.043, 064, 099, 116) were used. All three pMLST genes-based phylograms showed two major clusters similar to the phylogram generated from core-genome sequences, that is, separating p8025, pNDM-CIT, pPSP-75c, and p34983 plasmids from the remaining ones. Although the topologies of computed trees from pMLST CDS were not the same, the majority cluster can be further subdivided into two subgroups: one subgroup comprising plasmids pAKU_1, pR27 and pPstx_12_1, and another subgroup comprising pHCM1, pB71, p109-9, pF8475, pMAK1, pEQ1, pEQ2, and pO111 (fig. 3*B*).

In addition to comparative analysis of plasmid nucleotide sequences, we also performed comparative analysis at protein level. Clustering of all protein sequences from all examined plasmids resulted in 640 clusters of homologous proteins (supplementary fig. S1, Supplementary Material online). Out of these, 128 protein clusters, that is, highly similar proteins, were encoded by all examined plasmids. Hierarchical clustering based on the mere presence or the absence of protein grouped plasmids p8025, pNDM-CIT, pPSP-75c, and p34983 together, similar to the DNA sequence-based comparisons.

### Proteomic Analysis

**Fig. 4.—:**
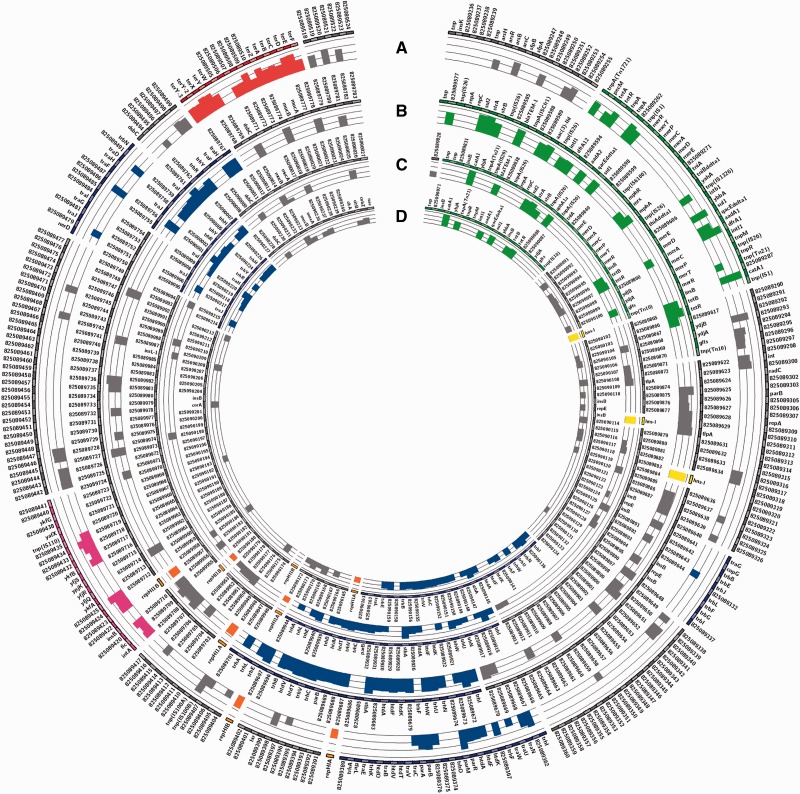
Protein expression of four completely sequenced plasmids present in *S*. Typhimurium F98 grown in LB broth supplemented with tetracycline at 24 °C for 20 h. (*A*) p8025, (*B*) pF8475, (*C*) p109-9 and (*D*) pB71. Histogram columns overlaid on the plasmid map indicate expressed genes/proteins. The height of each column indicates the quantity of each detected protein. Green, MDR region; blue, transfer regions; orange, replication region; pink, CP4 prophage; red, tellurium resistance region; gray, hypothetical proteins; yellow, H-NS-like protein.

Plasmids, especially those of high-molecular weight, are expected to affect the fitness of their host due to the costs associated with plasmid replication, maintenance, and protein expression. In the final experiment, we therefore analyzed protein expression of the genes located at pB71, p109-9, pF8475, and p8025. Such analysis was facilitated by the fact that having the full plasmid sequences, we were able to generate specific protein databases for protein MS. In total, 100 proteins (out of 175) from pB71, 104 (out of 200) from p109-9, 134 (out of 208) from pF8475, and 158 (out of 291) from plasmid p8025 were detected in *S.* Typhimurium grown in LB broth supplemented with tetracycline at 24 °C for 20 h (fig. 4, supplementary table S3, Supplementary Material online) and similar results were recorded also under the other culture conditions (supplementary table S3, Supplementary Material online). When gene expression was analyzed within the core-genome genes, 42–55%, 50–51%, 44–68%, and 41–45% out of 122 core-genome genes were expressed from plasmids pB71, p109-9, pF8475, and p8025, respectively, when grown in at least one out of the three slightly different culture conditions (supplementary table S3, Supplementary Material online). Genes encoding resistances to antibiotics belonged among those with the highest expression. *catA1*, *bla*_TEM_, *sul1*, *sul2*, *strA*, *strB*, *aphA1a*, *aadA1*, *dfrA1*, *aad(3)IId* genes were expressed under all experimental conditions including those performed in the absence of antibiotics. DNA-binding protein (H-NS-like protein) known to be involved in gene silencing (Doyle et al. 2007) was expressed from plasmids pB71, p109-9, and pF8475. In addition, genes coding for tellurium resistance and genes of CP4 prophage present in p8025 were also highly expressed under all experimental conditions tested. *repHI1A*, *repHI1B*, and genes coding for conjugational transfer were highly expressed from all four of the plasmids after 20 h growth at 24 °C but were only mildly expressed in the other two experiments, especially following the 6-day incubation at 4 °C.

### GC Content of Core- and Accessory-Genome Genes

Because H-NS- and H-NS-like proteins are involved in gene silencing of AT-rich genes (Lucchini et al. 2006; Dillon et al. 2010); finally, we analyzed the GC content of expressed and nonexpressed genes within the core-genome and within accessory genes of the pan-genome (table 1, supplementary table S4, Supplementary Material online). When we compared the GC content of the expressed and nonexpressed genes, either within the core-genome or accessory genes of pan-genome, no significant differences were found. On the other hand, the GC content of the genes forming the core-genome was always significantly lower in all four sequenced plasmids than the GC content of the genes forming the accessory genome. However, the lowest GC content was observed in the noncoding regions indicating that silencing could effectively occur, independent of GC content within genes.

**Table 1:** GC content in genes of core-genome, accessory genome and total GC content of noncoding sequences

	GC % in core-genome genes	GC % in accessory-genome genes	Total GC % in noncoding regions
p8025	45.71 ± 4.53^a^	54.32 ± 8.32	47.43
pF8475	45.88 ± 4.66	51.57 ± 8.74	43.85
p109-9	45.95 ± 4.66	49.77 ± 9.00	43.12
pB71	45.82 ± 4.62	48.97 ± 7.68	42.76

a Number represents the average GC content from all genes ± standard deviation.

A high average GC content in the genes forming the accessory genome and a high standard deviation indicated that distinct genes or regions with a high- and low-GC content might be present. We therefore viewed GC content of individual genes from the core-genome and accessory genome. As expected, a low variation was observed in the genes forming the core-genome. On the other hand, higher variation was observed among the accessory genes and genes forming the MDR region, that is, coding for resistance to antibiotics and mercury, represented those with the highest GC content. The only exception were *tet(B)* and *tetR* genes present in plasmids pB71, p109-9, and pF8475.

### Prediction of the Original Bacterial Host Species of IncHI1 Plasmid Sequences

The most likely original bacterial host for p109-9, pF8475, and pB71 plasmids was *Escherichia coli* because the frequency of 16-mers in the core-genome sequences was similar to their appearance in the genome of *E. coli*. However, the genes of the accessory genomes of p109-9, pF8457, and pB71 originated mainly from *Klebsiella oxytoca* (supplementary table S5, Supplementary Material online). The origin of p8025 was different from the previous three plasmids and its core genome was a mosaic of genes likely originating from *Shigella*, *Bacillus*, *Streptococcus*, *Haemophilus*, and *Desulfotalea*. On the other hand, the accessory genome of p8025 originated mostly from *Pseudomonas* (supplementary table S5, Supplementary Material online).

## Discussion

In this study, we determined the complete nucleotide sequences of four high molecular weight plasmids from the IncHI1 incompatibility group. Comparison of the complete nucleotide sequences of these plasmids showed that current IncHI1 plasmids are composed of an IncHI1 backbone and numerous insertions of mobile elements, MDR region in particular.

The MDR regions of all four plasmids contained up to five transposons such as Tn10 with tetracycline-resistance genes, Tn9 with chloramphenicol-resistance gene or Tn21 associated with mercury-resistance genes and Class 1 integrons (Holt et al. 2007; Dolejska et al. 2014). In p109-9, *bla*_TEM_-*strA*-*strB*-*sul1* genes were part of transposon Tn6029 which was also found in pHCM1 or pAKU_1. Tn6029-like transposon was detected also in pF8475 but IS26 upstream of *bla_TEM_* present in Tn6029 was replaced with ISCfr1 in this plasmid. A similar arrangement was described in pU302L in which IS26 was replaced with IS1294 (Chen et al. 2007). Tn6029 and related structures were identified not only in numerous antibiotic resistance plasmids (Holt et al. 2007) but also integrated in chromosomes of *E. coli* and *S. enterica* indicating an important role of Tn6029 and conjugative plasmids in dissemination the *bla*_TEM_*-sul2-strA-strB* antibiotic-resistance gene cluster among *Enterobacteriaceae* (Daly et al. 2005; Roy Chowdhury et al. 2015).

In the previous study, Holt et al. (2007) predicted the core-genome of IncHI1 plasmids to be 164 kbp based on sequence analysis of three IncHI1 plasmids. Our analysis comprising 15 IncHI1 plasmids reduced the predicted core-genome size to 123,370 bp encoding 122 genes. Comparative analysis at protein level revealed that 128 protein clusters were encoded by all investigated plasmids. Discrepancy between the number of shared genes and proteins was likely caused by different gene prediction strategy, especially in gene calling of short and/or hypothetical genes. The core-genome and proteome of examined plasmids included mainly genes necessary for replication and conjugal transfer. The size of approximately 123 kbp corresponds to the molecular weight of plasmids found in *Salmonella* from the preantibiotic era (Jones and Stanley 1992). Even though IS1 transposase was found in all examined plasmids, that is, it was classified as part of the core-genome, the IS1 element may have not been present in an ancestral IncHI1 plasmid backbone. Instead, IS1 could have been the first mobile element which inserted into IncHI1 backbone thus facilitating the insertions of additional sequences, although it cannot be excluded that ancestral IncHI1 plasmids already possessed transposase and/or genes coding for resistance to antimicrobial agents (Allen et al. 2010).

Independent of the sequences used for plasmid comparison, the constructed phylogenetic trees separated the plasmids into two distinct clusters. The first group consisted of p8025, pNDM-CIT, p34983, and pPSP-75C, that is, the plasmids >280 kbp originating from different genera such as *Salmonella*, *Citrobacter*, *Enterobacter*, and *Pantonea*. Interestingly, except for p34983, all these plasmids lacked the HCM1.78ac gene (*hns*-like gene). The second cluster comprised remaining plasmids and could be further subdivided into two subgroups. These subgroups corresponded with previously described Type 1 and Type 2 IncHI1 plasmids (Cain and Hall 2013), although the position of pHCM1 plasmid is equivocal. Cain and Hall (2013) clustered pHCM1 plasmid among Type 2 IncHI1 plasmids while our comparison based on core backbone sequences showed that pHCM1 belonged to Subgroup 1, although the branching was not statistically significant. However, when pMLST gene sequences were used, the pHCM1 plasmid clustered among Subgroup 2 IncHI1 plasmids, similar to the conclusions of Cain and Hall (2013). Clustering analysis based on the presence of particular gene or protein in the population cannot simulate the ancestral phylogenetic relationship. Unlike the phylogenetic tree-based approach, it can track recent genetic changes by horizontal gene transfer. Despite this, hierarchical clustering analysis confirmed that two different types of IncHI1 plasmids circulate within the population, however, further delineation of the major plasmid cluster was slightly different from nucleotide based comparison.

The GC content of core-genome genes indicated that they originate in species different from *Salmonella* for which a higher GC content than those found in IncHI1 core-genome is characteristic (McClelland et al. 2001). Low variation in the GC content of core-genome genes also indicated that IncHI1 plasmids had adapted to their original host during evolution.

On the other hand, a higher average GC content and a high variation in the GC content of accessory genes indicated their recent acquisition into the IncHI1 plasmid backbone. Although the average GC content of accessory genes was close to *Salmonella* GC content, at least the genes coding for antibiotic resistance likely do not originate from the original host of IncHI1 plasmid or from its current host. The high GC content rather points towards their origin in high GC content bacteria such as soil bacteria (Woolhouse et al. 2015), consistent with our prediction that substantial proportion of gene from accessory genome were likely derived from *Pseudomonas* or *Klebsiella*.

When we characterized the protein expression of IncHI1 plasmids, approximately half of the genes were expressed under any of the experimental conditions tested, irrespective of whether these belonged to the core-genome or accessory genome. Interestingly, genes responsible for resistance to chloramphenicol, β-lactam antibiotics, sulphonamide, streptomycin, spectinomycin, trimethoprim, neomycin, and tetracycline were constitutively expressed under all growth conditions including culture in antibiotic-free media. The likely explanation for this observation is that because most of these antibiotics inhibit proteosynthesis, inducible expression would not be feasible because the proteins conferring antibiotic resistance need to be expressed before the antibiotic can inactivate proteosynthesis. This is different from the inducible vancomycin resistance in *Enterococcus* sp. (Silva et al. 1998) when transient suppression of cell wall biosynthesis may allow bacterial cells to sense, induce and synthesize proteins necessary for their protection against vancomycin activity. High and reproducible expression of antibiotic-resistance genes also showed that despite several reports (Dillon et al. 2010; Doyle et al. 2007; Lucchini et al. 2006), the H-NS-like protein present in the majority of IncH plasmids does not silence the expression of recently acquired genes of the accessory genome in general. On the other hand, in agreement with expectations, proteins necessary for plasmid replication and conjugative transfer were highly expressed at 24 °C whereas their expression decreased at 4 °C.

## Conclusions

We have determined the complete sequences of four IncHI1 plasmids. Based on available sequences, we determined the core-genome of IncH1 plasmid consisting of 122 genes. Within the total IncHI1 plasmid pan-genome, each of sequenced plasmids contained an MDR region of different composition. IncHI1 plasmid genes responsible for resistance to antibiotics were constitutively expressed whereas the expression of genes necessary for plasmid replication, maintenance, and conjugative transfer was inducible and dependent on growth conditions.

## Supplementary Material

Supplementary files S1–S4, figure S1, and tables S1–S5 are available at *Genome Biology and Evolution* online (http://www.gbe.oxfordjournals.org/).

Supplementary Data
