# Supplementary material for: Antibiotic Resistance, Core-Genome and Protein Expression in IncHI1 Plasmids in Salmonella Typhimurium
Source: Genome Biol Evol. 2016 May 5;8(6):1661–71. doi: 10.1093/gbe/evw105 (PMC5390554; doi:10.1093/gbe/evw105)
Supplement: Supplementary Data [file supp_8_6_1661__index.html]

Antibiotic Resistance, Core-Genome and Protein Expression in IncHI1 Plasmids in Salmonella Typhimurium — Supplementary Data 

# Antibiotic Resistance, Core-Genome and Protein Expression in IncHI1 Plasmids in *Salmonella* Typhimurium

## Supplementary Data

files

- Supplementary Data - zip file
