# Supplementary figures and images for: Antibiotic Resistance, Core-Genome and Protein Expression in IncHI1 Plasmids in Salmonella Typhimurium
Source: Genome Biol Evol. 2016 May 5;8(6):1661–71. doi: 10.1093/gbe/evw105 (PMC5390554; doi:10.1093/gbe/evw105)

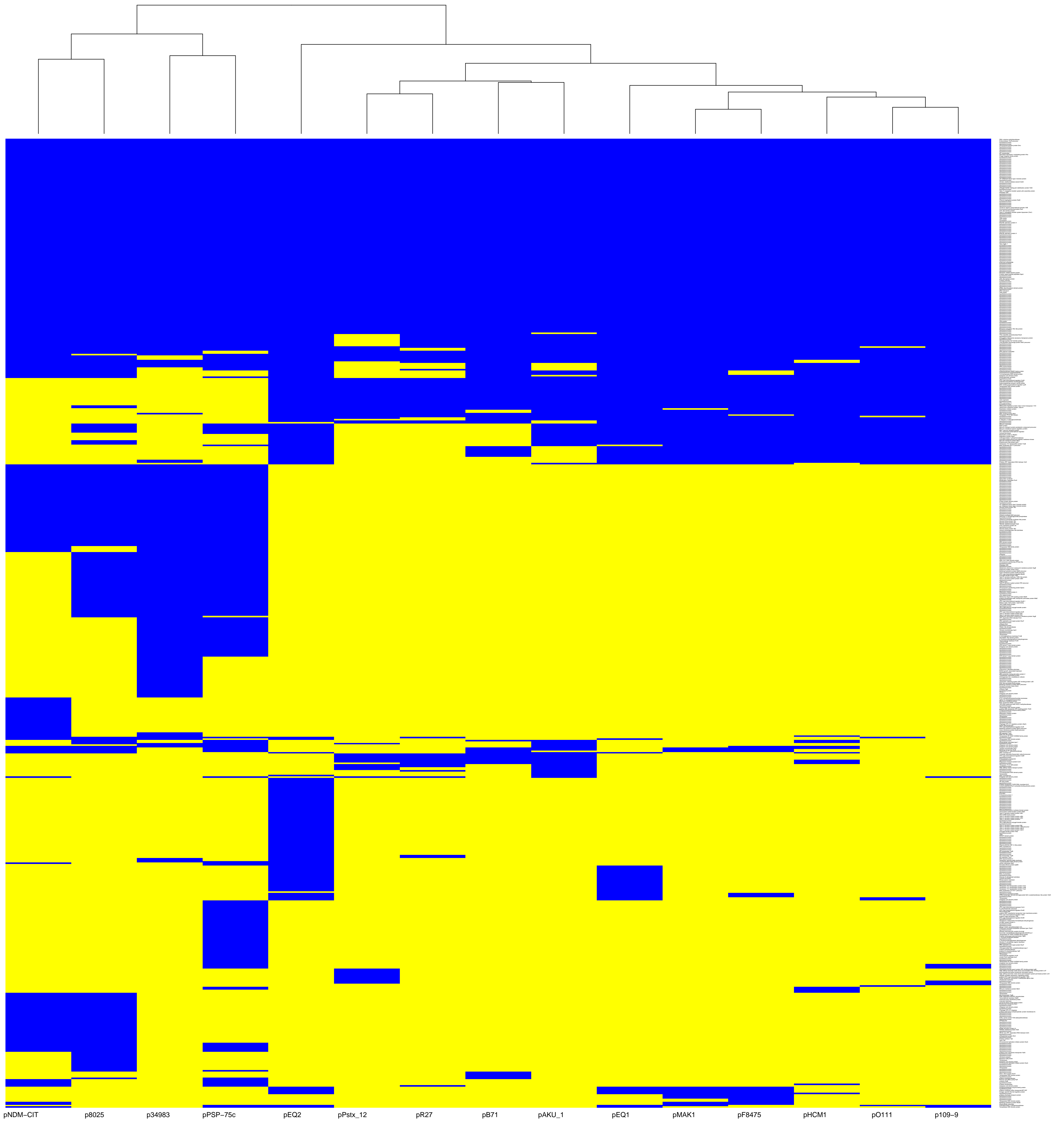

Supplement: Supplementary Data [file supp_evw105_suppl_data.zip › Figure_S1.pdf]
